# A Woman's Ideas of Peace

**Published:** 1918-12-28

**Authors:** 


### THE MATRONS' AND SISTERS' DEPARTMENT.

### A WOMAN'S IDEAS OF PEACE.

ONLY two or three months ago it looked as if this Christmas season of 1918 was likely to be one of the darkest in the history of our country. By contrast with those anticipations the festival shines with a light of heartfelt thanksgiving gladder than any this generation has tasted. There is little hilarity about. There is still extreme pressure of work and too few hands to accomplish it. There is very little money to spare, and very few luxuries to be bought. All that matters less than nothing, now that the background of our thoughts is freed from the tremendous nightmare of war. When the mind turns from old habit, often in the course of the day and night to the battlefields of France and Flanders, it is to contemplate a victorious army, meeting everywhere a frantically joyous reception, decked with flowers, half ashamed, half exultant, at the spontaneous outburst of gratitude their appearance evokes. For the moment it is more than enough to know that they suffer no longer. We taste the long unaccustomed joy of souls unburdened by a national sorrow and are content.

How strange it is to realise in this quiet transition time between peace and war that the last four years has revealed at last to mankind that the entire secret of statesmanship is summed up in the message of the angels "Peace on earth, good will towards men." It has taken men nearly two thousand years to find out that this is no pietistic maxim heralding the escape of a few elect souls from their wrathful Creator, but the assertion of a new principle without which the whole fabric of humanity crumbles surely to decay. Never since the first Christmas morning has universal peace seemed more nearly in the grasp of mankind than at the present. Never has the world come nearer to a recognition of the basic principles of liberty and justice, implied in the simple formula "Good will towards men."

It will be for the statesmen sent to office for the first time by the voice of men and women in unison to create the great reforms. It will be for individuals to see to the details, and make the reconstruction for which the nation waits a living reality. More and more clearly it becomes evident that women will have a big share in the task of carrying into effect the decisions of statesmen. And among women it is the nurses on whom will rest the burden of carrying into effect many priceless reforms which will bring a new note into family life.

Good will towards men. Avowedly it is this principle under which the Peace Conference begins the amazing labours which are to end war. To be faint-hearted and sceptical at this stage is for the onlookers to damp so far as may lie in their power the sacred fire which consumes the baser motives. Who can doubt it is from the aspirations of their several nations that the men chosen for this high duty will draw their strength? If ever in the darkest days we recognised in the hearts of our warriors in the field the steadfast staying-power which comes from God, let us not fail in the momentous days of the Peace Conference, nor doubt that the principle of Peace on Earth, which Christ Himself brought into the world, is within men's power to realise to-day.

Good will towards men. It is the secret of the nurse's vocation. It is the denial of base and ignoble aims, the surrender of life to deeds worth doing, the key to happiness. But shall we set a race of careworn women to train the new generation of probationers in the path of achievement? Can the overworked, underpaid nurse, whose future is ill-assured, whose health is undermined by labours which age her before her time, can such a woman be sent with any hope of success to bear the message of a larger hope? The answer is "Yes; it has been done, and it can still be done." Yes. But how much priceless energy has been wasted in the doing of it! How dearly has the nursing profession paid in lives and minds by setting its best members to fight with clogs on their feet, impeded in all their acts by conditions which sap their strength and vitiate their inner lives. Good will towards men. Yes. And good will towards nurses, also. Let us see to it that justice and liberty be the watchwords of the nursing service as its members gird themselves for the coming struggle to establish Peace on Earth

